# Examination of the Educational Needs of Home Visit Nurses: A Cross-Sectional Descriptive Study

**DOI:** 10.3390/ijerph18052319

**Published:** 2021-02-26

**Authors:** Kyuri Lee, Dukyoo Jung

**Affiliations:** College of Nursing, Ewha Womans University, Seoul 03760, Korea; kyurilee2501@gmail.com

**Keywords:** visiting nurses, home-visit nursing, long-term care, nursing education, needs assessment

## Abstract

This study aimed to identify educational needs and determine priorities in nursing care for home visit nurses providing services within the national long-term care insurance system in South Korea. This cross-sectional descriptive study assessed the educational needs of 92 home visit nurses. Participants’ characteristics were analyzed using percentages, averages, and standard deviations. Educational needs were calculated for participants’ current and required competency levels, utilizing the paired *t*-test, Borich’s educational needs formula, and the locus for focus model. Four main categories were identified as top priorities: (1) health assessment, (2) cognitive function care, (3) disease management, and (4) medication management. The study’s findings could support the development of appropriate and responsive education and training programs for home-visit nurses—as it reflects the actual needs of this group.

## 1. Introduction

Home visit nursing is regarded as a cost-effective means of care management that satisfies the desires of elderly adults who wish to remain in their homes amid the world’s aging population problems [1,2]. South Korea (hereafter referred to as Korea) has one of the fastest-aging populations. The proportion of adults aged >65 years has increased by 14.4%, already indicating an aged society, and this is expected to accelerate, creating a super-aged society by 2025 [3]. Individuals experience challenges in self-care as they age, including comorbidity, frailty, and functional decline [4,5]. Moreover, physical challenges can lead to an increased risk of mental health difficulties such as depression or anxiety [6]. The demand for home-based nursing services is rising as physical and mental health disorders are becoming increasingly prevalent in the aged population.

In order to address population aging, the Korean government implemented the elderly long-term care insurance system in July 2008. This system involves social insurance (older adults are eligible regardless of income) and provides long-term care services to individuals who cannot conduct their daily activities alone because of old age or geriatric disease [7]. Home visit nursing is the only medical service provided by this Korean insurance system for older adults living in the community. Home visit nursing is an interactive process between nurses, their clients, and clients’ families in the home setting, with consequences that could include improvements in the clients’ health [8]. Evidence suggests that home visit nursing services exert a positive effect on physical and mental health [9,10], and have been reported to contribute to reductions in medical costs [11]. Recently, the aging in place concept (which refers to the ability to remain safe in one’s own home and community [12]) has been embraced by many countries’ long-term care policies. Despite the current old-age health strategy and the growing need for home-visit nurses (HVNs), the education system for home visit nursing under long-term care insurance is insufficient as existing education programs focus primarily on hospital-based, rather than community-based nursing [13,14,15].

According to Orem’s self-care deficit theory, nursing is needed when individuals and families are limited in their ability to provide continuous self-care for the promotion and maintenance of health [16]. Therefore, based on the theory, nurses need to determine their clients’ self-care deficits and their own roles as nurses [17]. The competency of nurses is a major factor in high-quality care and role implementation [18]. Spencer and Spencer [19] stated that competency was an underlying personal characteristic related to effective performance. HVNs require high competency levels, as home nursing involves a broad range of professional knowledge and skills. Home nursing services range from basic to complex, and HVNs provide required care autonomously, based on the diverse needs of individuals. Their services include: assessment of clients’ general health status and home environment, management of underlying health conditions, health promotion, and implementing curative interventions [20,21]. Hence, although HVNs have clinical experience in hospitals, the competency of HVNs providing care in community settings requires improvement [22]. Unfortunately, there is no current mandatory education and training for HVNs in Korea. The development of such programs is necessary—and an understanding of the educational needs of HVNs is required in order to facilitate effective educational development [23].

A need is defined as a gap between what should be and what is [24]. Needs assessment is the systematic process of specifically identifying what learners need most, and using that information to inform the directions and goals of education with limited resources [25]. Borich’s needs assessment [26], a needs analysis model, uses a formula that weighs the required competency level (RCL; i.e., what should be) and the discrepancy between the RCL and the present competency level (PCL; i.e., what is). In addition, Mink, Shultz, and Mink’s locus for focus (LF) model [27] can be used to prioritize educational needs via quadrants—separated by the average value of the two-level difference and the RCL—to supplement the Borich needs assessment, with which it can be difficult to determine top priorities [28].

This study aimed to identify priorities in educational needs for HVNs using Borich’s needs assessment and the locus for focus model. The findings herein could inform future strategies and guide the effective development of education for visiting nurses.

## 2. Materials and Methods

### 2.1. Study Aim and Design

We conducted a descriptive cross-sectional study to identify HVNs’ educational needs and priorities with regard to home nursing services based on long-term care insurance.

### 2.2. Participants

The participants were HVNs in Korea. The inclusion criteria were: the provision of home-visit nursing services based on the Long-term Care Insurance Act for the Elderly, and more than six months of work experience as an HVN. The exclusion criterion was the absence of the provision of nursing services directly to insurance beneficiaries. The sample size was calculated using G*Power 3.1.9 (University of Dusseldorf, Dusseldorf, Germany). In a paired t-test, a minimum sample size of 90 was required, with an effect size of 0.30, a significance level of 0.05, and statistical power of 0.80. Considering a 10% dropout rate, we recruited 99 HVNs. Of these, seven participants were excluded because of missing or insincere responses. Thus, 92 HVNs participated in the study.

### 2.3. Data Collection

HVNs were recruited from long-term care institutions and an online community for HVNs. After obtaining contacts for long-term care institutions registered on the insurance website, one of the researchers contacted institution directors to describe the study and obtained permission to post recruitment advertisements that included a URL linking to the survey. Because of the spread of COVID-19, data were collected via an online survey created using Google Forms, from 20 April to 17 May 2020. HVNs who met the eligibility criteria and wished to participate in the study voluntarily clicked on the URL and were supplied with recruitment materials.

### 2.4. Instrument

The questionnaire collected information on participants’ general characteristics and educational needs. To assess educational needs, the perceived PCL and RCL for home nursing were measured using an instrument developed for the study. In total, 90 preliminary questions were extracted following a review of the literature [18,29,30,31,32,33]. The internal validity of the instrument was examined by five experts, including one gerontological nursing professor, one gerontological nurse practitioner, one home care nurse specialist, and two HVNs serving as directors. One item with an item-content validity index (I-CVI) under 0.80 was deleted, and one item (“nail care”) was added. The resultant instrument comprised 19 categories and 90 subcategories, and the summed score was 0.97. The PCL and RCL for each question were measured using a five-point Likert scale ranging from 1 (very low) to 5 (very high). Higher perceived PCL scores and lower perceived RCL scores indicated greater educational needs. The internal consistency of the instrument was examined using Cronbach’s α. Cronbach’s α for the overall instrument was 0.99. Cronbach’s α for each of the PCL and RCL questions was 0.99. Cronbach’s αs for the individual categories ranged from 0.73 to 0.94.

### 2.5. Data Analysis

All statistical analyses were performed by one of the researchers, using IBM SPSS Statistics, version 23.0 (IBM, Armonk, NY, USA). Participants’ characteristics were analyzed using percentages, averages, and standard deviations. Educational needs were analyzed utilizing the Borich needs assessment and the LF model. Borich’s formula is as follows:

(1) $$\mathrm{Borich}’s\;\mathrm{needs}\;\mathrm{score}\;=\;\frac{\sum \left(\mathrm{RCL}-\mathrm{PCL}\right)\times \bar{\mathrm{RCL}}}{n}$$

**Figure 1:**
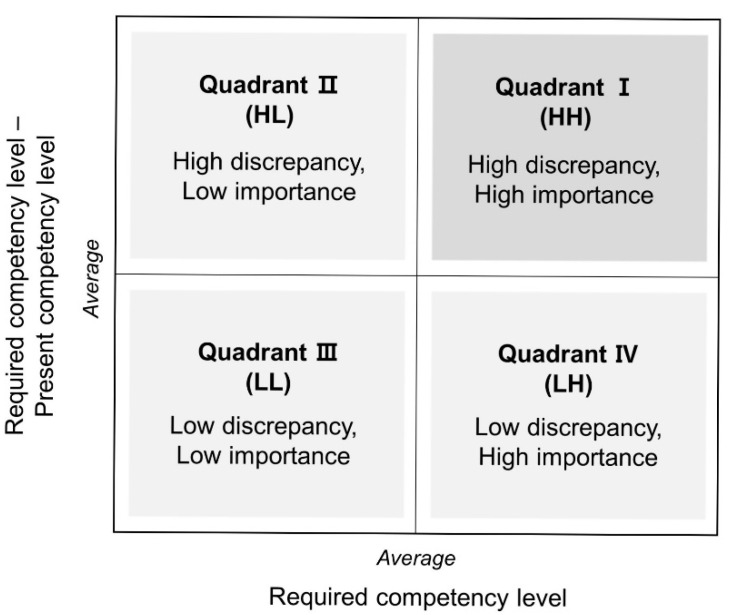
Locus for focus (LF) model.

According to Borich’s formula, educational need [25] was calculated by multiplying the sum of the difference between the PCL and RCL (∑RCL-PCL) by the mean of the RCL and dividing the result by the total number of cases (*n*). In addition, the LF model was used to determine high priority [27]. The LF model consists of two coordinate axes: the horizontal axis represents the RCL, and the vertical axis represents the difference between the RCL and PCL. As shown in Figure 1, Quadrant 1 is the highest priority area, with two levels of difference (that is, two levels of inconsistency), and the RCL are higher than average (HH quadrant) [27].

Top-priority categories were identified throughout the prioritization process in the needs assessment proposed by Cho [28]. The process was as follows: (1) identify the overall trend through paired *t*-tests examining differences between the PCL and RCL; (2) identify educational needs using the Borich needs assessment; (3) identify the number of educational needs priorities belonging to the HH quadrant, using the LF model; (4) count the number of categories belonging to the HH quadrant in the LF model and determine the top rank of Borich needs that fits the count; and (5) determine the highest and second-highest priority groups by checking the redundancy between the high-ranking categories of the Borich needs assessment and the HH quadrant of the LF model.

### 2.6. Ethical Considerations

The study adhered to the ethical guidelines of the Helsinki Declaration, and was approved by the institutional review board at the university with which the researchers were affiliated (IRB No. 202003-0006-03). Researchers provided details of the study on the first screen that appeared when accessing the online survey. Informed consent was obtained from the nurses, who clicked a consent button prior to beginning the survey. Data will be stored for three years after the conclusion of the study, after which all data will be deleted in accordance with the National Bioethics and Safety Act.

## 3. Results

### 3.1. Participant Characteristics

Table 1 shows the characteristics of the 92 HVNs who participated in the study. Participants’ mean age was 42.42 years, and most were women (95.7%). More than 70% of participants had a bachelor’s degree in nursing or higher level of education. Participating HVNs had an average of 2.96 years’ experience in home-visit nursing and 16.3% had an advanced practice nurse certificate. Over 80% of HVNs indicated that education was a necessity for them.

### 3.2. Priority Categories for Educational Needs in Home Visit Nursing

**Figure 2:**
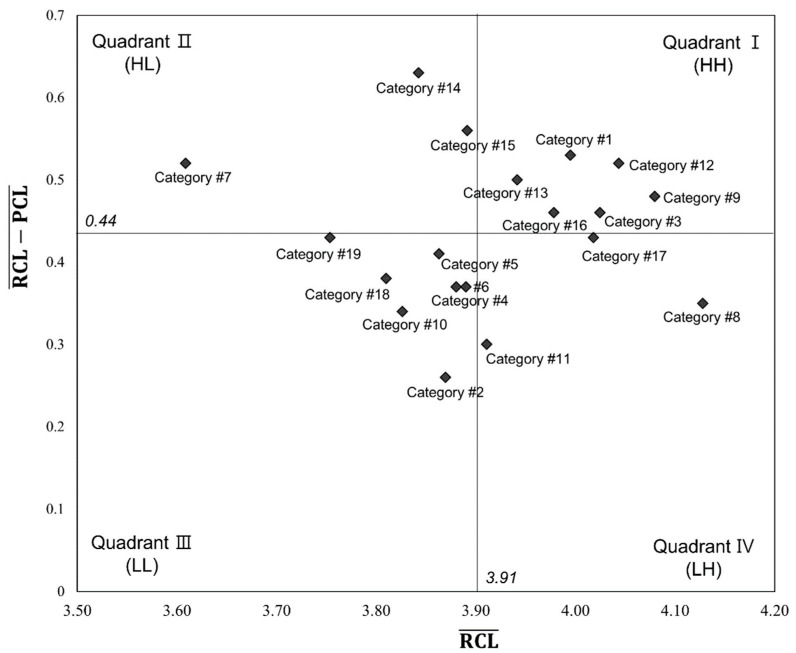
The LF model of home visit nursing.

Table 2 shows the average PCL and RCL for all categories. There were statistically significant differences between the two levels in all 19 categories and in the overall average; the PCL was lower than the RCL. Table 2 and Figure 2 show educational need priorities, identified by applying the Borich needs assessment and LF model, respectively. The results of checking the redundancy between the high-ranking categories of two models showed that the following four categories were identified as top priorities in home visit nursing: health assessment, cognitive function care, geriatric disease management, and medication management (Table 3).

### 3.3. Top-Priority Categories for Educational Needs in Home Visit Nursing

Table 4 shows the top-priority subcategories, identified using the same prioritization process. In total, 14 of 90 categories were identified as top priorities; of these, six were included in the health assessment category.

## 4. Discussion

This study examined the top priorities in HVNs’ educational needs, using the Borich’s needs assessment and LF models, in accordance with the five stages of the prioritization process [28]. The aim of identifying educational needs was to contribute to the effective development of education and training programs that reflect the situation in the field.

According to the study’s results, HVNs perceived gaps between the PCL and RCL in home nursing. The PCL was significantly lower than the RCL in all categories and items; this finding was similar to those of a previous study [34]. Learners feel the need for education when there are differences between the PCL and RCL [35]. Therefore, we considered the difference significant, as >80% of participants felt the need for education in home nursing, and 90.2% were willing to participate in such education. As a number of nursing education programs focus on acute care in hospitals, HVNs have identified a paucity of education in home-based nursing care [13,14,15]. Therefore, the results indicate that the development of educational programs to strengthen HVNs’ competencies in practice is imperative.

Through duplicate verification via the Borich needs score and LF models, the top-priority categories for educational needs were identified as: health assessment, cognitive function care, disease management of the elderly, and medication management.

Borich’s needs score for the health assessment category was highest, even in the top-priority category group; this finding is similar to that of a previous study conducted with hospital nurses [36]. Advanced assessment skills are crucial to identify patient needs and provide proper nursing interventions [37]. According to the nursing approach outlined in Orem’s self-care theory, collecting data through assessment is the first step toward detecting problems that need to be addressed [16]. In particular, HVNs recognize the importance of assessment—they provide hands-on care regularly in patients’ homes. However, the lack of segmented health assessment training in home-based settings could have affected the need for education.

The second-highest priority category was cognitive function nursing, and the cognitive training program was identified as the highest priority item. The proportion of beneficiaries with dementia was 31.3% in 2009, at the beginning of the insurance system, but has recently increased by 57.2% [38]. As dementia prevalence increases with the policy of pursuing aging in place, the demand for education and training in cognitive function care is also expected to increase [39]. In addition, given the limitations of drug therapy in dementia and the need for non-drug interventions because of drug side effects, it appears that the need for cognitive training programs has increased. Therefore, in designing a dementia education curriculum for HVNs, educators should focus on cognitive training methods that are applicable in home-care settings.

The third highest priority category was disease management; care for cardiovascular disease, mental health, and neurological disease for elderly adults were identified as subcategories of the highest priority. As heart disease is a major cause of death in the aged population, and the number of cases continues to rise [40], management of cardiovascular disease is considered important by HVNs, leading to a correspondingly high educational need. Further, the need for education in the management of mental disorders was ranked highest in the study. Older adults are particularly vulnerable to mental disorders; first, because they are socially isolated compared to the younger population, and second, because loss, health problems, or functional decline can give rise to depression and anxiety, which can lead to suicidal thoughts [41]. Mental illness management can therefore be regarded as critical care in home health nursing. Moreover, HVNs expressed a high educational need in the management of neurological diseases. It has been reported that the proportion of beneficiaries who experienced a stroke accounted for 25.8% of the total number of beneficiaries [38]. In addition, neurological diseases, such as stroke and Parkinson’s disease, require continuing physical rehabilitation [42]. Therefore, educational programs should be developed and implemented with a focus on disease management—the area identified for which HVNs have the greatest educational need, at present.

The last top-priority category was medication management, with oral medication management as the item of highest priority. These findings are consistent with previous studies showing that oral medication administration and recording were the highest educational needs for HVNs [34]. Insurance beneficiaries have been reported to display an average of three or more chronic disorders [38], and those with multiple morbidities have to take multiple drugs. As polypharmacy in older adults involves a high risk of poor compliance, misuse, and side effects [33,43], the understanding and management of the major drugs associated with underlying medical conditions can be regarded as an important part of home nursing. Therefore, education programs should include content (such as interventions) to enhance drug adherence and prevent duplicate use.

Of the high-priority subcategories identified, exercise programs, communication, and the prevention and management of suicidal behavior were not included among the top-priority categories. Prevention and management of suicidal behavior ranked highest in the safety management category. Even though nurses play a key role as gatekeepers in preventing suicide, there is a dearth of suicide prevention programs for caregivers, including HVNs [44]. Therefore, the provision of education to prevent and cope with suicide in older adults is crucial.

Exercise programs ranked highest in the physical training category. The assessment committee of long-term care insurance rates individuals according to the time period required for long-term care. The proportion of beneficiaries with rating levels 1 and 2 (who are generally bedridden and unable to move without assistance) has decreased, while that of beneficiaries with rating levels 3, 4, and 5 (who can generally move and walk) has increased from 48.1% in 2010 to 71.5% in 2017 [45]. This trend seems to have influenced HVNs’ educational needs. Prior studies have reported that exercise programs exerted a positive effect on physical and cognitive improvements and mental health [46,47]. Accordingly, continuing education regarding exercise methods, such as muscle strengthening, flexibility exercise, or exercise using simple equipment at home, is required.

Communication ranked highest in the education and counseling category. This finding is consistent with those of a previous study conducted with long-term care hospital nurses [36]. Improving communication skills, which are fundamental tools for therapeutic relationships with patients [48], is essential, particularly for HVNs; they provide education, counseling, and emotional support while visiting beneficiaries and family caregivers regularly for at least an hour. Further research is required to identify learning needs for highly specific items, e.g., investigating educational needs for pre-entry, entry, working, and termination phase communication skills.

According to the results of the current study, educational needs were lower for physician-ordered therapeutic nursing services than for other nursing services. This might be due to an increase in the number of care recipients with ratings of level 3 or higher, requiring less-invasive treatment. Furthermore, current policies in aging countries focus on prevention and management rather than treatment-oriented care. In fostering client and caregiver self-care and wellness within home environments, HVNs should function in various roles [17] and take a more comprehensive view with a range of skilled healthcare services [21]. Thus, educators should design and develop education and training programs to enhance HVNs’ competency based on the current findings. These findings have indicated what HVNs truly need to learn in order to provide superior home visit nursing services, as well as increase the learners’ own participation rate and satisfaction.

The current study was subject to some limitations. For example, the study used convenience sampling, and further research with larger sample sizes and random sampling is required to establish generalizability. Additionally, self-reported data could include response bias. Moreover, the difference in educational needs according to recipients’ long-term care rates was not considered. Finally, the needs assessment questionnaire may need to be shortened or modified for more accurate assessment, as the internal consistency for the overall instrument was too high. This may suggest the presence of redundant items [49], although Cronbach’s α was acceptable for individual categories.

## 5. Conclusions

This study systematically investigated the educational needs of HVNs caring for older people experiencing functional decline, which has not previously been well-explored. The results confirmed the top-priority categories and subcategories for educational needs in home visit nursing services. The current study can be used to support and facilitate the further development of continuous education and training programs for HVNs, as it provides an overall understanding of their professional educational needs. Future studies can expand on these findings by focusing and reflecting on educational needs when developing program content and curricula.

## Figures and Tables

**Table 1:** Participant characteristics (*N* = 92).

Variable	*n* (%)	Mean (*SD*)
Age (years)		42.42 (9.44)
Female	88 (95.7)	
Education		
College degree	19 (20.7)	
Bachelor’s degree	50 (54.3)	
Master’s degree	17 (18.5)	
Doctoral degree	6 (6.5)	
Years of experience in nursing (except home visit nursing)		9.65 (7.67)
<5	29 (31.5)	
5–9.9	24 (26.1)	
≥10	25 (38.1)	
Years of experience in home visit nursing		2.96 (2.88)
<1	23 (25.0)	
1–4.9	50 (54.3)	
≥5	19 (20.7)	
Position in institution		
Operator ^1^	35 (38.0)	
Staff	57 (62.2)	
Licensure		
RN	77 (83.7)	
APRN	15 (16.3)	
Need for education		
Not necessary at all	0	
Not necessary	2 (2.2)	
Neutral	14 (15.2)	
Necessary	41 (44.6)	
Very necessary	35 (38.0)	
Intention to participate in education		
No	9 (9.98)	
Yes	83 (90.2)	

^1^ Home visiting nurses can serve as operators of institutions, which involves sole proprietorship. SD = standard deviation. RN = registered nurse. APRN = advanced practice registered nurse.

**Table 2:** Educational needs in home visit nursing (*N* = 92).

Category	PCL	RCL	Gap	*t (p)*	Cohen’s *d*	Borich	
	Mean (SD)	Mean (SD)	Mean (SD)			Needs Score	Ranking
1. Health assessment	3.46 (0.56)	4.00 (0.56)	0.53 (0.61)	8.33 (<0.001)	0.87	2.12	3
2. Hygiene care	3.61 (0.71)	3.87 (0.59)	0.26 (0.58)	4.39 (<0.001)	0.45	1.02	19
3. Physical training	3.57 (0.66)	4.02 (0.64)	0.46 (0.77)	5.73 (<0.001)	0.60	1.85	8
4. Respiratory care	3.45 (0.75)	3.86 (0.79)	0.41 (0.86)	4.55 (<0.001)	0.48	1.58	12
5. Gastrointestinal care and nutritional management	3.51 (0.72)	3.88 (0.68)	0.38 (0.70)	5.10 (<0.001)	0.54	1.46	13
6. Urinary and bowel management	3.52 (0.73)	3.89 (0.71)	0.37 (0.63)	5.57 (<0.001)	0.59	1.43	16
7. Other tube care	3.09 (0.97)	3.61 (0.99)	0.52 (1.01)	4.95 (<0.001)	0.51	1.88	7
8. Skin care	3.78 (0.71)	4.13 (0.57)	0.35 (0.68)	4.91 (<0.001)	0.51	1.44	14
9. Medication management	3.60 (0.75)	4.08 (0.67)	0.48 (0.79)	5.82 (<0.001)	0.61	1.91	6
10. Laboratory test management	3.48 (0.73)	3.83 (0.67)	0.34 (0.69)	4.79 (<0.001)	0.49	1.32	17
11. Comfort care	3.61 (0.62)	3.91 (0.62)	0.30 (0.63)	4.65 (<0.001)	0.48	1.19	18
12. Cognitive function management	3.53 (0.75)	4.04 (0.59)	0.52 (0.77)	6.49 (<0.001)	0.68	2.10	4
13. Disease management	3.44 (0.73)	3.94 (0.61)	0.50 (0.68)	7.01 (<0.001)	0.74	1.96	5
14. Hospice care	3.22 (1.01)	3.84 (0.81)	0.63 (0.95)	6.34 (<0.001)	0.66	2.47	1
15. Family nursing	3.33 (0.83)	3.89 (0.71)	0.56 (0.81)	6.66 (<.001)	0.69	2.20	2
16. Education and counseling	3.52 (0.77)	3.98 (0.67)	0.46 (0.81)	5.42 (<0.001)	0.57	1.83	9
17. Safety management	3.59 (0.72)	4.02 (0.63)	0.43 (0.73)	5.68 (<0.001)	0.59	1.74	10
18. Resources and environment management	3.43 (0.73)	3.81 (0.65)	0.38 (0.81)	4.46 (<0.001)	0.47	1.44	14
19. Administrative, legal, and ethical work	3.33 (0.91)	3.75 (0.76)	0.43 (0.94)	4.37 (<0.001)	0.48	1.60	11
Total Average	3.48 (0.58)	3.91 (0.53)	0.44 (0.55)	7.66 (<0.001)	0.80		

PCL = present competency level, RCL = required competency level, SD = standard deviation.

**Table 3:** Priority categories for educational needs (*N* = 92).

Category	Borich Ranking	LF model Quadrant	Top Priority	Second Priority
Hospice care	1	HL		✔
Family nursing	2	HL		✔
Health assessment	3	HH	✔	
Cognitive function care	4	HH	✔	
Disease management	5	HH	✔	
Medication management	6	HH	✔	
Physical training		HH		✔
Education and Counseling		HH		✔

LF = locus for focus.

**Table 4:** Top-priority subcategories for educational needs (*N* = 92).

Category	Subcategory	Borich	LF Model Quadrant
Health assessment	Cardiovascular system assessment	2.54	HH
Health assessment	Mental health assessment	2.40	HH
Health assessment	Respiratory system assessment	2.35	HH
Health assessment	Skin assessment	2.28	HH
Health assessment	Musculoskeletal system assessment	2.21	HH
Health assessment	Gastrointestinal system assessment	2.11	HH
Cognitive function care	Cognitive training program	2.56	HH
Disease management	Cardiovascular diseases of the elderly	2.62	HH
Disease management	Mental health disorders	2.24	HH
Disease management	Neurological diseases of the elderly	2.11	HH
Medication management	Oral medication management	2.19	HH
Safety management	Prevention and management of suicidal behavior	2.56	HH
Physical training	Exercise programs	2.41	HH
Education and Counseling	Communication	2.31	HH

## Data Availability

Not applicable.
